# A Rare Case of Paclitaxel and/or Trastuzumab Induced Acute Hepatic Necrosis

**DOI:** 10.1155/2015/825603

**Published:** 2015-10-29

**Authors:** Hiren Mandaliya, Pinky Baghi, Amy Prawira, Mathew K. George

**Affiliations:** Tamworth Rural Referral Hospital, Dean Street, Tamworth, NSW 2340, Australia

## Abstract

Paclitaxel induced mild derangement of liver functions including bilirubin, alkaline phosphatase, and AST has been infrequently noticed in clinical trials. Contrary to Paclitaxel, hepatocellular injury, hepatitis, and liver tenderness are common laboratory and clinical findings with Trastuzumab. However, hepatic failure/necrosis secondary to Paclitaxel or Trastuzumab has never been reported in literature. A 62-year-old lady, previously healthy, was treated with adjuvant therapy for left breast stage II, high grade invasive ductal carcinoma which was node negative, oestrogen receptor negative, progesterone receptor positive, and HER2 receptor positive. After modified radical mastectomy and axillary clearance, she finished four cycles of Doxorubicin/Cyclophosphamide chemotherapy and then commenced on Paclitaxel/Trastuzumab combination chemotherapy. Within twelve hours of first dose of Paclitaxel/Trastuzumab therapy, patient required hospital admission for acute onset respiratory failure. Patient died within 36 hours of therapy and autopsy was suggestive of acute hepatic necrosis without any other significant findings. Detailed investigations were not carried out as event was quick with rapid deterioration. There was no history of prior liver pathology/injury and preliminary investigations for major organ involvement were unremarkable. As per our knowledge, Paclitaxel and/or Trastuzumab induced acute hepatic necrosis has never been reported in literature before, hence difficult to predict.

## 1. Introduction

Paclitaxel (Taxol) is increasingly being used to treat ovarian and breast cancer [1, 2]. Trastuzumab (anti-HER2 therapy) has been used in both metastatic and adjuvant breast cancer and improves response rate, time to progression, and overall survival, hence approved for combination therapy with Taxol as well. Trastuzumab is a humanised monoclonal immunoglobulin G1 kappa antibody that binds to the extracellular membrane domain of HER2 and inhibits the proliferation and survival of HER2-dependent tumours. Adjuvant therapy of Paclitaxel and Trastuzumab has been used in numerous trials, has been shown to have a high response rate, and is currently one of the standards of care. Well-known adverse effects from Paclitaxel are myelosuppression and neurotoxicity. Uncommonly, Paclitaxel induced mild derangement of liver functions with elevated enzymes had been noted in clinical trials. Reversible cardiotoxicity due to Trastuzumab is well known but severe congestive heart failure occurred in 0.6% of patients treated in HERA (BIG-01-01) trial. Commonly, hepatocellular injury/hepatitis and liver tenderness were noted with Trastuzumab; however hepatic failure has not been noted.

## 2. Case Presentation

We report the case of 62-year-old lady who was diagnosed with carcinoma breast, who underwent modified radical mastectomy and axillary clearance. Histopathology was suggestive of stage II, high grade invasive ductal carcinoma, 25 mm sized tumour, which was axillary node negative, ER negative, PR positive, and HER2 (SISH) positive. She achieved her menopause 12 years ago. She did not have any major health issues and remained well functionally. Adjuvant chemotherapy AC (Doxorubicin/Cyclophosphamide) was planned for her, followed by Paclitaxel/Trastuzumab. Her baseline pretreatment cardiac function, liver functions, full blood count, renal functions, and electrolytes were within normal range. Patient was commenced on AC chemotherapy around one month following her curative surgery. Four cycles of AC chemotherapy were given at 3 weekly intervals and prior to each chemotherapy cycle she was reviewed for toxicities assessment including blood tests and tolerance to chemotherapy as per protocol. She travelled satisfactorily without any major chemotherapy related toxicities. On completion of initial phase of her chemotherapy, weekly Paclitaxel and three times weekly Trastuzumab had been scheduled as per plan. Prior to this commencement, she was reviewed and her blood was unremarkable. She had a first dose of Paclitaxel/Trastuzumab. Within 12 hours of administration of Paclitaxel/Trastuzumab infusion, patient presented to emergency unit with acute respiratory distress. Patient's chest X-ray showed features of pulmonary oedema/acute respiratory distress syndrome. Initial diagnosis was cardiac failure/acute respiratory failure of unknown cause. Laboratory investigations on arrival which included full blood count, liver functions, renal functions, electrolytes, and troponin were inconclusive. She was not neutropenic and her septic screen was negative. ECG and transthoracic echocardiogram were normal. She did not have any history of alcohol abuse or hepatitis. She had never used alternative medicines, herbal preparations, or any hepatotoxic agents. Her prechemotherapy blood was unremarkable as well. She deteriorated very quickly despite initial acute resuscitation. She was admitted to CCU (Coronary Care Unit) where she was put on respiratory support as her condition declined so rapidly. She was unstable to perform any imaging because rapidity at which she progressed did not allow it. Patient died within 36 hours of receiving the first dose of Paclitaxel/Trastuzumab. Surprisingly, autopsy showed acute hepatic/liver necrosis.

## 3. Discussion

As acute hepatic necrosis which was proven on autopsy in our case, we think Paclitaxel and/or Trastuzumab are the most likely culprit. Points which favour these are acute onset following exposure to these agents, no liver injury/toxicities from previous chemotherapeutic agents (Doxorubicin/Cyclophosphamide), and no prior history of hepatitis or excess of alcohol; however, it is difficult to prove. According to the validated drug-induced hepatotoxicity scales [3–6], certain criteria should be met in order to prove that a liver injury is caused by the medication such as temporal relation between drug and liver injury, reversal of liver abnormality on cessation of drug, and exclusion of other determinants of liver injury and reproducibility. Hepatic injury attributed to Paclitaxel is considered to be infrequent in clinical studies [7]. In patients with normal baseline liver function who are treated with Paclitaxel, transient elevations of alkaline phosphate, transaminases (particularly AST), and bilirubin have been reported in about 5 to 20 percent of cases [7]. Hepatic toxicity is not dose-dependent and prolonged exposure to taxanes is not associated with cumulative hepatic toxicity. Trastuzumab related reactions generally occur with the first dose, possibly during or immediately following the infusion. Severe and rarely fatal adverse reactions including acute respiratory distress syndrome are known to occur. Trastuzumab related cardiotoxicity is most often manifested by an asymptomatic decrease in left ventricular ejection fraction (LVEF) and less often by clinical heart failure [8]. Trastuzumab induced hepatotoxicity requiring discontinuation of the drug has been reported as well [9].

Acute liver failure caused by an idiosyncratic drug reaction has a mortality rate of over 80 percent without liver transplantation [10–12]. However in our case it is difficult to prove that it was Paclitaxel and/or Trastuzumab that caused the hepatic necrosis as the patient died before further investigations could be initiated. We strongly recommend that liver enzymes levels must be closely monitored in patients receiving Trastuzumab and Paclitaxel.
